# Intense, stable and excitation wavelength-independent photoluminescence emission in the blue-violet region from phosphorene quantum dots

**DOI:** 10.1038/srep27307

**Published:** 2016-06-06

**Authors:** Shuaipeng Ge, Lisheng Zhang, Peijie Wang, Yan Fang

**Affiliations:** 1 The Beijing Key Laboratory for Nano-Photonics and Nano-Structures, Department of Physics, Capital Normal University, Beijing 100048, China

## Abstract

Nanoscale phosphorene quantum dots (PQDs) with few-layer structures were fabricated by pulsed laser ablation of a bulk black phosphorus target in diethyl ether. An intense and stable photoluminescence (PL) emission of the PQDs in the blue-violet wavelength region is clearly observed for the first time, which is attributed to electronic transitions from the lowest unoccupied molecular orbital (LUMO) to the highest occupied molecular orbital (HOMO) and occupied molecular orbitals below the HOMO (H-1, H-2), respectively. Surprisingly, the PL emission peak positions of the PQDs are not red-shifted with progressively longer excitation wavelengths, which is in contrast to the cases of graphene and molybdenum disulphide quantum dots. This excitation wavelength-independence is derived from the saturated passivation on the periphery and surfaces of the PQDs by large numbers of electron-donating functional groups which cause the electron density on the PQDs to be dramatically increased and the band gap to be insensitive to the quantum size effect in the PQDs. This work suggests that PQDs with intense, stable and excitation wavelength-independent PL emission in the blue-violet region have a potential application as semiconductor-based blue-violet light irradiation sources.

Black phosphorus, a typical two-dimensional Van der Waals crystal with a puckered hexagonal structure, has attracted a great deal of attention due to its unique structure and anisotropic electron-transport, thermal transport and optical properties^1^,^2^,^3^. Compared with the planar structure of graphene^4^,^5^ the characteristic puckered layered structure of phosphorene provides a larger space for lithium storage, which leads to a higher energy density in the charged state and allows phosphorene to be a promising anode material for lithium ion batteries^6^,^7^,^8^. Furthermore, black phosphorus is a semiconductor material, but the small band gap (0.3 eV) limits its applications as a photoluminescence (PL) emitter^9^,^10^. Phosphorene exhibits a PL emission at 855 nm because its band gap is expanded to approximately 1.45 eV due to the confinement effect^11^. But the band gap of phosphorene is still too small and the PL emission is too weak for it to be useful as a PL material. By analogy with the PL emission behaviours of graphene quantum dots^12^,^13^ the band gap of phosphorene quantum dots (PQDs) should be further expanded and the PL emission positions should be blue-shifted because of the size-tuned optical response. This implies that an opportunity exists to create a new type of quantum dot material with PL emission. However, few studies on the PL emission of PQDs have been reported so far. On the other hand, because of the activity of black phosphorus in ambient conditions, the conventional methods to fabricate graphene, molybdenum disulphide and other two-dimensional Van der Waals crystal quantum dots, such as the hydrothermal route^14^,^15^,^16^ acidic exfoliation^17^ and electrochemical strategies^18^ are not applicable methods for PQD materials^19^,^20^. It was reported that black phosphorus quantum dots could be prepared in N-methylpyrrolidinone (NMP) solvent by an ultrasonic exfoliation route^19^. But these quantum dots are not available as a PL material because NMP solvent exhibits strong emission^21^ and it has not been proved if this quantum dot material itself can efficiently photoluminesce.

In this work, the PQD samples were synthesized by pulsed laser ablation of a bulk black phosphorus target in diethyl ether. Pulsed laser ablation of solid materials in liquids is a clean and orientable technique for fabricating nanomaterials, such as metal and semiconductor nanoparticles, and carbon-related nanomaterials^22^,^23^,^24^. The intense and stable PL peaks of the PQDs are found in the blue-violet wavelength region. It is noted that the PL emission peak positions of the PQDs are not red-shifted with the increase of excitation wavelengths, which is in contrast to the cases of graphene and molybdenum disulphide quantum dots. Because of the activity of black phosphorus, the periphery and surfaces of the PQDs are saturatedly passivated by substituents from cracked diethyl ether by laser ablation. This leads to the dramatic increase of the electron density on the periphery and surfaces of the PQDs. This causes the band gap of the PQDs to be relatively insensitive to the quantum size effect^12^,^25^. PQDs with intense, stable and excitation wavelength-independent PL emission in blue-violet region have a potential application as semiconductor-based blue-violet light irradiation sources.

## Results and Discussion

**Figure 1:**
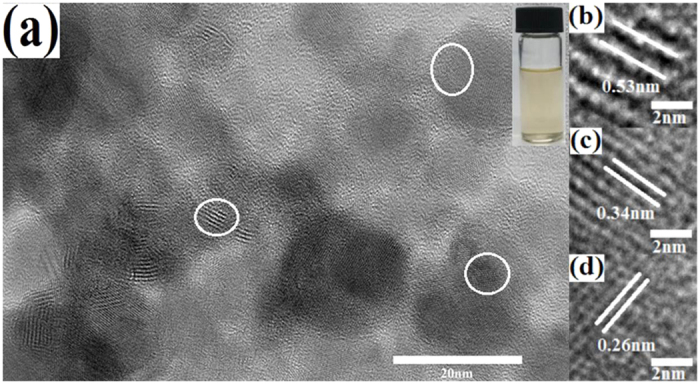
(**a**) A TEM image of the PQDs and a photo of PQD solution under visible light in the inset. HRTEM images of circles in (**a**) with lattice parameters of (**b**) 0.53 nm, (**c**) 0.34 nm and (**d**) 0.26 nm, respectively.

Figure 1a shows a TEM image of the PQDs that were prepared by pulsed laser ablation of a bulk black phosphorus target. More micrographs about the morphology and structure of PQDs are given in supplementary information (Fig. S1a–e). Statistically, the average size of the PQDs is ~7 nm, and most of the particles are less than 10 nm in size (Fig. S1f). The localized lattice structures of PQDs are clearly observed. The corresponding HRTEM images of PQDs in Fig. 1b–d give lattice parameters of 0.53 nm, 0.34 nm and 0.26 nm, which can be ascribed to the (020), (021) and (040) planes of black phosphorus crystal, respectively^26^,^27^ confirming that the PQDs still retain the puckered hexagonal structure after pulsed laser ablation.

**Figure 2:**
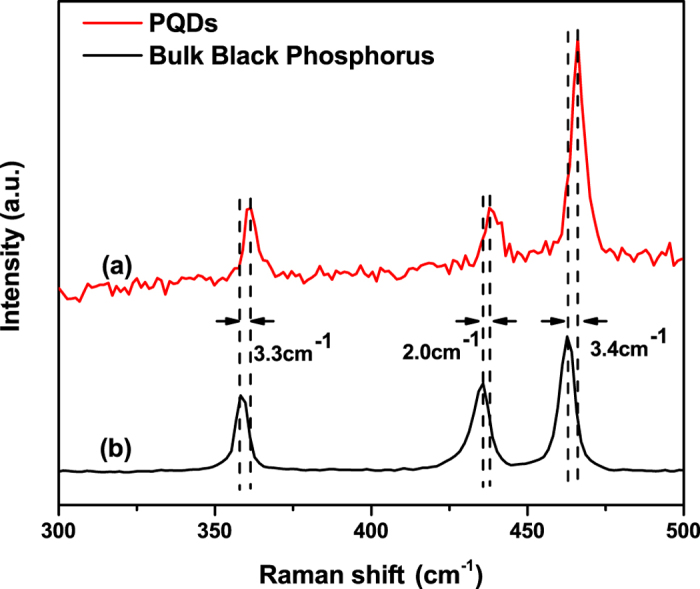
Raman spectra of PQDs (curve **a**), and the bulk black phosphorus crystal (curve **b**).

As shown in Fig. 2, PQDs also have three characteristic Raman peaks of the bulk black phosphorus crystal, which are located at 361.5 cm^−1^, 437.8 cm^−1^ and 466.1 cm^−1^, respectively. The first peak is ascribed to out-of-plane phonon modes (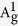), and the latter two to in-plane modes (B_2g_ and 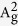)^28^,^29^ indicating that PQDs still exhibit the unique puckered hexagonal layered structure. Furthermore, the change of the intensity ratio of 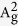 and 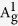 from the bulk black phosphorus crystal (1.7) to the PQDs (2.3) demonstrates that PQDs possess a few-layer structure^28^. Additionally, the 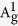, B_2g_, and 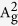 vibrational modes of PQDs, compared with that of the bulk black phosphorus crystal, are red-shifted by approximately 3.3 cm^−1^, 2.0 cm^−1^ and 3.4 cm^−1^, respectively. This red-shift can be attributed to the change of van der Waals interlayer interaction, similar to the cases of graphene and molybdenum disulphide quantum dots with low thickness and small size^18^,^30^.

**Figure 3:**
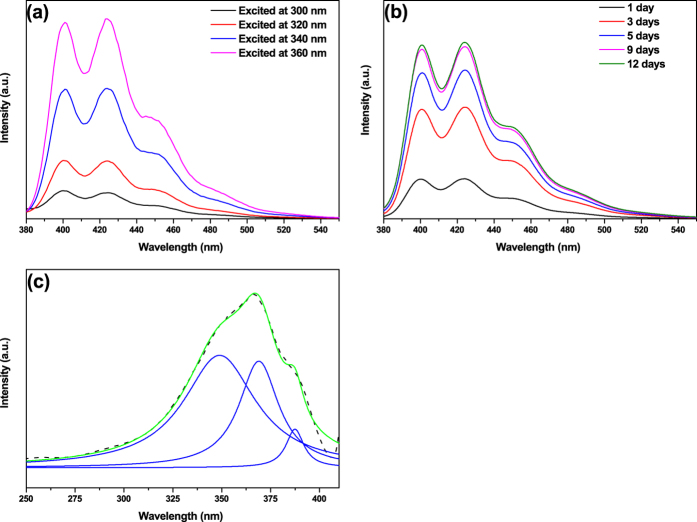
(**a**) PL emission spectra of PQDs excited with varying wavelengths from 300 nm to 360 nm. (**b**) PL emission spectra of PQDs excited at 360 nm measured from 1st to 12th day. (**c**) The measured PL excitation spectrum of PQDs by monitoring the PL peak at 424 nm (dashed line), sum of Lorentzian fittings (green line), and three Lorentzian fittings (blue lines).

Figure 3a shows the PL emission spectra of the PQDs in the blue-violet wavelength region, which are excited with varying wavelengths from 300 nm to 360 nm. It is necessary to clarify that these PL emissions are originated from PQDs, rather than from diethyl ether, the passivation molecules, because the emission of diethyl ether that is mainly located in the ultraviolet region is very weak so as to be ignored, compared to the PL emission spectra of PQDs (Supplementary Information, Fig. S2). The peak positions of the PL emission of PQDs remain unshifted as the excitation wavelength changes (Fig. 3a). This excitation wavelength-independent PL behaviour of PQDs is in contrast to the cases of graphene and molybdenum disulphide quantum dots, in which their PL emission peak positions are always shifted depending on the excitation wavelengths. Additionally, the intensities of the PL peaks increase with progressively longer excitation wavelengths (Fig. 3a). The similar increase in intensity can also be observed with time prolonged (Fig. 3b) because the aggregation of PQDs with time further reduces non-radiative decay of PQDs leading to the enhancement of PL emission^31^. The intensities are stable after 12 days with PL quantum yield of 11.92%, which is higher than that of reported graphene (4–11%)^25^,^32^ and molybdenum disulphide (1.3 or 2.6%)^15^,^33^ quantum dots. In summary, all these PL properties indicate that the PQDs have the potential to be a good candidate to build nanostructured materials for intense, stable and excitation wavelength-independent semiconductor-based blue-violet light irradiation sources, and extend the range of application to optical and electronic devices.

The PL emission mechanism of PQDs can be further understood by the PL excitation spectrum in Fig. 3c. The PL excitation spectrum (dashed line) of PQDs is measured by monitoring the PL emission peak at 424 nm. The summary of Lorentzian fittings (green line) gives a reasonable approximation of the measured PL excitation spectrum, and consists of three excitation bands (blue lines) at 349 nm (~3.55 eV), 369 nm (~3.36 eV) and 387 nm (~3.20 eV), respectively. The energy difference between the two PL excitation peaks at 349 nm (~3.55 eV) and 369 nm (~3.36 eV) is ~0.19 eV. Notably, this value almost equals the energy difference between the two PL emission peaks at 400 nm (~3.10 eV) and 424 nm (~2.92 eV). Similarly, the energy difference between the two PL excitation peaks at 369 nm (~3.36 eV) and 387 nm (~3.20 eV) is also nearly equals that of the two PL peaks at 424 nm (~2.92 eV) and 448 nm (~2.77 eV). These certify that the PL mechanism of the PQDs involves three emission transitions from the lowest unoccupied molecular orbital (LUMO) to the highest occupied molecular orbital (HOMO) and occupied molecular orbitals below the HOMO (H-1, H-2), respectively. Thus, the model of the ground state which contains three energy states can be given. Actually, the H-2, H-1 and HOMO states are determined by the sizes and the structures of the periphery and surfaces of the PQDs. Therefore, it is necessary to further study the passivation process (i.e., the formation process of the PQDs).

In the process of preparing PQD samples by pulsed laser ablation in diethyl ether, a micro area on the surface of the bulk black phosphorus crystal rapidly absorbs sufficient energy and is heated to a high temperature. As a result, the phosphoric clusters on the laser ablated micro area break out of the surface boundary of the bulk black phosphorus crystal and crack into nanoscale few-layer phosphorene domains, just a skeleton of PQDs, by overcoming the Van der Waals forces. Simultaneously, the diethyl ether molecules are cracked by laser ablation to passivate the surfaces and edges of the nanoscale phosphorene domains to form electron-donating functional groups. Finally, the PQDs that consist of few-layer nanoscale phosphorene domains and electron-donating functional groups on the periphery and surfaces are obtained.

**Figure 4:**
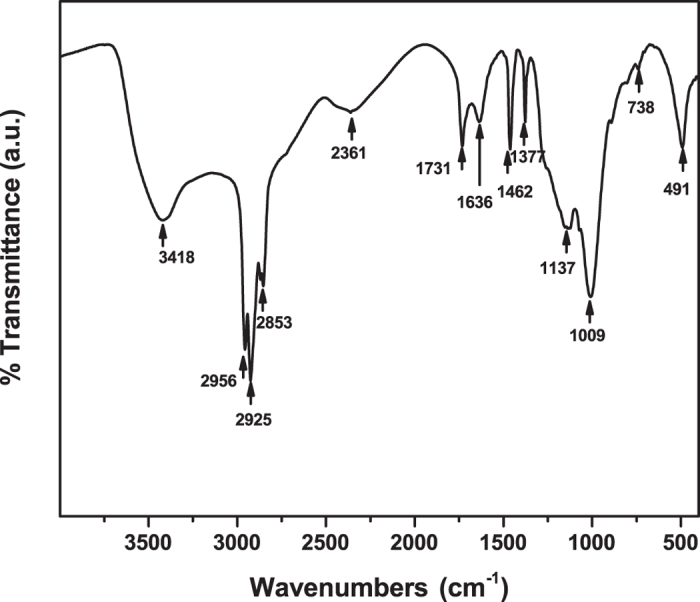
FTIR spectrum of PQDs.

The passivation effect can be further evidenced by the FTIR spectrum of the PQDs (Fig. 4). The strong peak at 1009 cm^−1^ is attributed to the stretching vibration of the P-O-C bonds. The absorption peak at 1137 cm^−1^ is ascribed to the stretching vibration of the P=O bonds. In addition, the bending and stretching vibrations of P-OH are found at 1731 cm^−1^ and 2361 cm^−1^, respectively, suggesting the possible formation of electron-donating functional groups on the periphery and surfaces of the PQDs.

As mentioned above, the peak positions of PL emission remain unshifted as the excitation wavelength changes from 300 nm to 360 nm (Fig. 3a). This typical excitation wavelength-independent PL behaviour of the PQDs is in contrast to the case of graphene quantum dots, in which the PL emission peaks can be shifted with excitation wavelengths due to the quantum size effect. Because of the activity of black phosphorus, the surfaces and edges of the PQDs are easily and rapidly passivated, even saturatedly passivated, by large amount of functional groups (P-O-C, P=O, and P-OH etc.), including electron-donating groups. This passivation on the PQDs causes the enhancement of capability to donate electrons, which benefits the electron-transfer from functional groups to nanoscale phosphorene domains, and leads to the dramatic increase of the electron density on the periphery and surfaces of the PQDs. This sufficient passivation exerts a significant influence on the energy states of the H-2, H-1 and HOMO levels, for instance, causes the increase of electron energy of ground-state, which plays a role in the PL mechanism of the PQDs. As a result, HOMO, H-1 and H-2 levels of the PQDs are raised, and the band gaps become small, similar to the case of increasing the size of the PQDs to reduce the band gap. With the number of functional groups increased, the passivation could be saturated. Not only is the PL emission strongly enhanced but also the band gap becomes relatively insensitive to the quantum size effect as the HOMO, H-1 and H-2 levels are raised. The electron-donating functional groups play a leading role in controlling the PL emission of the PQDs in wavelength and intensity. Consequently, the PL peak positions of the PQDs show typical excitation wavelength-independence, indicating that PQDs with intense and stable PL emission in the blue-violet region will bring more opportunities for their applications in the field of semiconductor-based irradiation light sources.

## Conclusions

Nanoscale PQDs with few-layer structure were fabricated by pulsed laser ablation of a bulk black phosphorus crystal. The typical lattice fringes with parameters of 0.53 nm, 0.34 nm and 0.26 nm that are ascribed to the (020), (021) and (040) planes of the black phosphorus crystal, respectively, are clearly observed in the PQDs, indicating that the PQDs still retain the puckered hexagonal structure. The ratio of 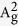 and 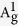, and the red-shifts of 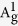, B_2g_, and 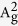 modes of the PQDs demonstrate that the PQDs possess a few-layer structure and small size. Three intense, stable PL peaks of the PQDs are found at 400 nm, 424 nm and 448 nm in the blue-violet region for the first time, which are attributed to electronic transitions from LUMO to HOMO, H-1 and H-2. The intensities of the PL peaks increase with progressively longer excitation wavelengths. Furthermore, the intensities of the PL peaks also increase with time, and stabilize with PL quantum yield of 11.92%. The PL emission peak positions of the PQDs show excitation wavelength-independence because the saturated passivation of the PQDs by sufficient functional groups causes the band gap to be relatively insensitive to the quantum size effect. As the result, the electron-donating functional groups play a leading role in controlling the PL emission of the PQDs in wavelength and intensity. All of these properties suggest that PQDs can be applied as semiconductor-based blue-violet light irradiation sources and will be a new type of quantum dot material with PL emission.

## Methods

The black phosphorus crystals were purchased from Smart Elements and stored in a dark glass dryer filled with Ar gas. Diethyl ether (99.0%) was purchased from Sinopharm Chemical Reagent.

The PQD solution was prepared in diethyl ether by a Nd:YAG pulsed laser with a wavelength of 1064 nm and a repetition rate of 5 Hz; the energy was 30 mJ pulse^−1^ and the pulse width was 6 ns. A target of bulk black phosphorus was placed at the bottom of a vessel with diethyl ether, and the pulsed laser was used to ablate target for twenty minutes to obtain the PQDs. The samples were kept in a sealed glass vial.

The size distribution and structure of the PQDs were observed by transmission electron microscopy (TEM) and high resolution transmission electron microscopy (HRTEM) (Model JEOL, JEM2100). Raman spectra were recorded using a Renishaw H13325 spectrophotometer with excitation at 532 nm (1.0 mW). The PQD solution was dropped onto an ultrathin carbon-coated holey carbon support film with 200 mesh copper grid for TEM measurement, and onto a clean Si substrate for Raman measurement. A Fourier transform infrared (FTIR) spectrum was obtained on a Nicolet 6700 FTIR spectrometer. The PL and PL excitation spectra of the PQDs were measured with a Hitachi F-7000 spectrophotometer. Absolute quantum yield was measured on a fluorescence lifetime and steady state spectrometer (Edinburgh Instrument, F920, with an integrating sphere). All measures were carried out at room temperature.

## Additional Information

**How to cite this article**: Ge, S. *et al.* Intense, stable and excitation wavelength-independent photoluminescence emission in the blue-violet region from phosphorene quantum dots. *Sci. Rep.* **6**, 27307; doi: 10.1038/srep27307 (2016).

## Supplementary Material

Supplementary Information

## References

[b1] Wang X. *et al.* Highly anisotropic and robust excitons in monolayer black phosphorus. Nat. Nanotechnol 6, 517–521 (2015). 25915195 10.1038/nnano.2015.71

[b2] Qiao J. *et al.* High-mobility transport anisotropy and linear dichroism in few-layer black phosphorus. Nat Commun 5, 4475 (2014). 25042376 10.1038/ncomms5475 PMC4109013

[b3] Liu T. H. & Chang C. C. Anisotropic thermal transport in phosphorene: effects of crystal orientation. Nanoscale 24, 10648–10654 (2015). 26024364 10.1039/c5nr01821h

[b4] Kvashnin A. G., Chernozatonskii L. A., Yakobson B. I. & Sorokin P. B. Phase diagram of quasi-two-dimensional carbon, from graphene to diamond. Nano Lett 2, 676–681 (2014). 24437392 10.1021/nl403938g

[b5] Rao C. N. R., Sood A. K., Subrahmanyam K. S. & Govindaraj A. Graphene: the new two-dimensional nanomaterial. Angew Chem Int Ed 42, 7752–7777 (2009). 10.1002/anie.200901678 19784976

[b6] Park C. M. & Sohn H. J. Black phosphorus and its composite for lithium rechargeable batteries. Adv Mater 18, 2465–2468 (2007).

[b7] Li W., Yang Y., Zhang G. & Zhang Y. W. Ultrafast and directional diffusion of lithium in phosphorene for high-performance lithium-ion battery. Nano Lett 3, 1691–1697 (2015). 25664808 10.1021/nl504336h

[b8] Zhao S., Kang W. & Xue J. The potential application of phosphorene as an anode material in li-ion batteries. J Mater Chem A 44, 19046–19052 (2014).

[b9] Zhang S. *et al.* Extraordinary photoluminescence and strong temperature/angle-dependent Raman responses in few-layer phosphorene. ACS Nano 9, 9590–9596 (2014). 25188827 10.1021/nn503893j

[b10] Gan Z. X. *et al.* Tunable photoluminescence from sheet-like black phosphorus crystal by electrochemical oxidation. Appl Phys Lett 2, 021901 (2015).

[b11] Liu H. *et al.* Phosphorene: An unexplored 2D semiconductor with a high hole mobility. ACS Nano 4, 4033–4041 (2014). 24655084 10.1021/nn501226z

[b12] Jin S. H. *et al.* Tuning the photoluminescence of graphene quantum dots through the charge transfer effect of functional groups. ACS Nano 2, 1239–1245 (2013). 23272894 10.1021/nn304675g

[b13] Zhu S. *et al.* Investigating the surface state of graphene quantum dots. Nanoscale 17, 7927–7933 (2015). 25865229 10.1039/c5nr01178g

[b14] Pan D., Zhang J., Li Z. & Wu M. Hydrothermal route for cutting graphene sheets into blue-luminescent graphene quantum dots. Adv Mater 6, 734–738 (2010). 20217780 10.1002/adma.200902825

[b15] Wang Y. & Ni Y. Molybdenum disulfide quantum dots as a photoluminescence sensing platform for 2,4,6-trinitrophenol detection. Anal Chem 15, 7463–7470 (2014). 25001878 10.1021/ac5012014

[b16] Aboulaich A. *et al.* One-pot noninjection route to CdS quantum dots via hydrothermal synthesis. Appl Mater Inter 5, 2561–2569 (2012). 10.1021/am300232z 22509818

[b17] Peng J. *et al.* Graphene quantum dots derived from carbon fibers. Nano Lett 2, 844–849 (2012). 22216895 10.1021/nl2038979

[b18] Li Y. *et al.* An electrochemical avenue to green-luminescent graphene quantum dots as potential electron-acceptors for photovoltaics. Adv Mater 6, 776–780 (2011). 21287641 10.1002/adma.201003819

[b19] Zhang X. *et al.* Black phosphorus quantum dots. Angew Chem Int Ed 12, 3653–3657 (2015). 10.1002/anie.201409400 25649505

[b20] Sun Z. *et al.* Ultrasmall black phosphorus quantum dots: synthesis and use as photothermal agents. Angew Chem 39, 11581–11586 (2015). 10.1002/anie.201506154 26296530

[b21] Gan Z. X. *et al.* Quantum confinement effects across two-dimensional planes in MoS_2_ quantum dots. Appl Phys Lett 106, 233113 (2015).

[b22] Mafune F., Kohno J., Takeda Y. & Kondow T. Formation of gold nanonetworks and small gold nanoparticles by irradiation of intense pulsed laser onto gold nanoparticles. J. Phys. Chem. B. 107, 12589–12596 (2003).

[b23] Zeng H., Cai W., Li Y., Hu J. & Liu P. Composition/Structural evolution and optical properties of ZnO/Zn nanoparticles by laser ablation in liquid media. J. Phys. Chem. B. 109, 18260–18266 (2005). 16853349 10.1021/jp052258n

[b24] Chen G. X. *et al.* Preparation of carbon nanoparticles with strong optical limiting properties by laser ablation in water. J. Appl. Phys 95, 1455–1459 (2004).

[b25] Tang L. *et al.* Deep Ultraviolet photoluminescence of water-soluble self-passivated graphene quantum dots. ACS Nano 6, 5102–5110 (2012). 22559247 10.1021/nn300760g

[b26] Sun J. *et al.* Formation of stable phosphorus-carbon bond for enhanced performance in black phosphorus nanoparticle-graphite composite battery anodes. Nano Lett. 8, 4573–4580 (2014). 25019417 10.1021/nl501617j

[b27] Lee H. U. *et al.* Stable semiconductor black phosphorus (BP)@titanium dioxide (TiO2) hybrid photocatalysts. Scientific Reports 5, 8691 (2015). 25732720 10.1038/srep08691 PMC4346807

[b28] Lu W. *et al.* Plasma-assisted fabrication of monolayer phosphorene and its Raman characterization. Nano Res 7, 853–859 (2014).

[b29] Wu J. *et al.* Identifying the crystalline orientation of black phosphorus using angle-resolved polarized Raman spectroscopy. Angew Chem Int Ed 8, 2366–2369 (2015). 10.1002/anie.201410108 25611334

[b30] Stengl V. & Henych J. Strongly luminescent monolayered MoS_2_ prepared by effective ultrasound exfoliation. Nanoscale 8, 3387–3394 (2013). 23467444 10.1039/c3nr00192j

[b31] Gao M. X. *et al.* A surfactant-assisted redox hydrothermal route to prepare highly photoluminescent carbon quantum dots with aggregation-induced emission enhancement properties. Chem. Commun 49, 8015–8017 (2013). 10.1039/c3cc44624g 23903411

[b32] Sun Y. P. *et al.* Quantum-sized carbon dots for bright and colorful photoluminescence. J. Am. Chem. Soc 128, 7756–7757 (2006). 16771487 10.1021/ja062677d

[b33] Ha H. D., Han D. J., Choi J. S., Park M. & Seo T. S. Dual role of blue luminescent MoS_2_ quantum dots in fluorescence resonance energy transfer phenomenon. Small 10, 3858–3862 (2014). 24976217 10.1002/smll.201400988

