# Supplementary material for: Intense, stable and excitation wavelength-independent photoluminescence emission in the blue-violet region from phosphorene quantum dots
Source: Sci Rep. 2016 Jun 6;6:27307. doi: 10.1038/srep27307 (PMC4893668; doi:10.1038/srep27307)
Supplement: Supplementary Information [file srep27307-s1.pdf]

Supplementary Information

Intense, stable and excitation wavelength-independent photoluminescence emission in the blue-violet region from phosphorene quantum dots

Shuaipeng Ge, Lisheng Zhang, Peijie Wang and Yan Fang*

The Beijing Key Laboratory for Nano-Photonics and Nano-Structures, Department of Physics, Capital Normal University, Beijing 100048, China
***Corresponding author: Yan Fang, E-mail: fangyan@cnu.edu.cn**

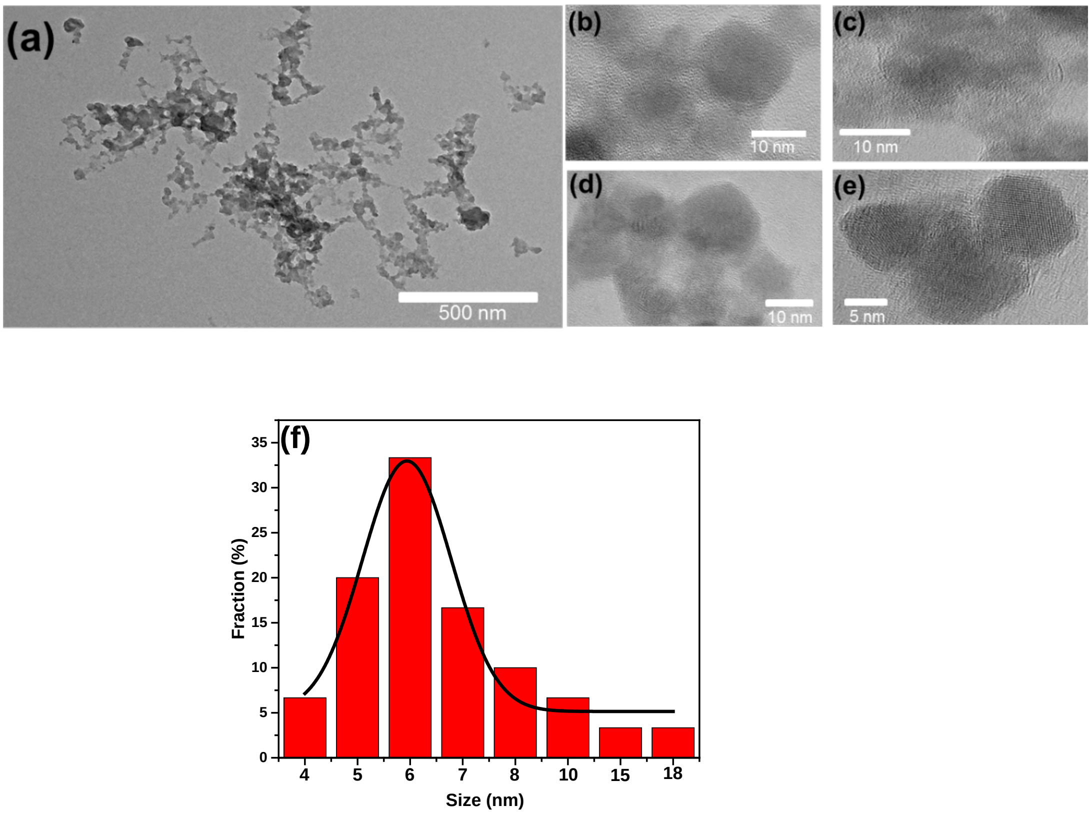

Fig. S1 (a) A TEM image of the PQDs at lower magnification. (b), (c), (d), (e) HRTEM images. (f) Diameter distribution of the PQDs.

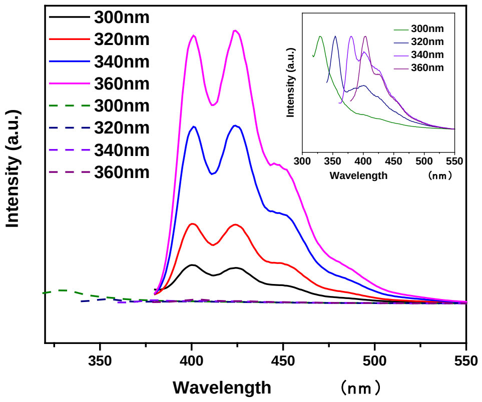

Fig. S2 PL emission spectra of PQDs (solid lines) and diethyl ether solvent (dashed lines) excited with varying wavelengths from 300 nm to 360 nm (the inset is the normalized PL emission spectra of diethyl ether).
